# Concept Animation - a potential instructional scaffolding

**DOI:** 10.15694/mep.2018.0000153.1

**Published:** 2018-07-24

**Authors:** Faraz Khurshid, Babu Noushad, Ingrid A. E Spanjers, Jamila Al-Darwashi

**Affiliations:** 1 University of Buraimi; 2 Department of Educational Development and Research & Graduate School of Health Professions Education

**Keywords:** Graphic Medicine, Comics, Visual Metaphors, Multimedia Learning, Constructivism, Cognitive loads, Concept cartoon, Cognitive schema, Self-explanation principle, Pharmacokinetics, Dose-related toxicity, Zone of proximal development

## Abstract

This article was migrated. The article was marked as recommended.

Concept animation - the graphical array of pictures accompanied by text as speech balloons, can help to improve learner’s comprehension from basic to advanced levels concepts. When the process of concept comprehension is not facilitated, the learner may come in a transitional state of misperception and understanding, that can restrain their learning to a surface approach. The basic science concepts learned at the inception of a Health Sciences program play a vital role towards the development of higher-order thinking and problem-solving aptitude in the subsequent years. Hence, it is important to facilitate meaningful learning of core concepts and principles in difficult basic science disciplines, like Pharmacology. This work reports our experiences of employing concept animations as a ‘visual aid’ instructional strategy to simplify pharmacology concepts to undergraduate Optometry students. The fundamental ideas of drug pharmacokinetics, pharmacodynamics and side effects were transformed into concept animations. The effects of these concept animations are explained by using Vygotsky’s ‘zone of proximal development, Mayer’s cognitive theory of multimedia learning and cognitive load theory.

## Overview

Concept development is a multistep learning process of knowledge acquisition that often becomes challenging and frustrating for learners. When the command on core concepts is improper and imprecise, the learners always find difficulty in grasping the progressive level big ideas that may limit cognition to a lower level of rote memorization. Undergraduate students studying the core concepts and principles of basic sciences recurrently experience this situation. Teaching innovations foster learning strategies to effectively support the process of knowledge acquisition without confining the knowledge to a primitive cognition level. For instance, multimedia learning can improve students understanding of a concept and topic by synchronous representations of verbal and visual information ( Mayer, 2002).

Graphic medicine is an example of multimedia learning and teaching innovation to foster student’s learning strategies. It has been utilized as an important pedagogical instrument and effective cognitive and metacognitive strategy to reinforce optometry student’s learning experience of basic sciences subject such as pharmacology ( Khurshid & Noushad, 2017). The graphic medicine guided approach of animating concepts via a series of diagrams placed in succession led us to ‘Concept animation’. The purpose of this strategy is to facilitate understanding of advanced level core concepts using diagrams and comics.

## Background

The ideology is a reflection of the concept cartoon, a strategy devised in 1992. Concept cartoon blends visual stimulus/images with key points or information as dialogues presenting a central idea or a core concept.  It functions as a powerful stimulus for learners to focus their attention on the meaningful description of the concept ( Fensham, Gunstone & White, 2013). It was an effort to develop innovative teaching and learning strategy on the constructivist aspects of the learning process with considerable novelty value. The two important phases of constructivism model included ‘Elicitation’ and ‘restructuring’. Elicitation helps students to make their ideas explicit through discussion or writing. While restructuring provides them, the chance to recognize, evaluate and reorganise their ideas to construct it further provided the opportunities. Concept cartoon brings together the elicitation of ideas with the restructuring of the knowledge in classroom teaching ( Keogh & Naylor, 1999).

This method showed a significant level of learner’s motivation and attention; it helped to sustain student’ level of interest and facilitate information sharing and discussion. Moreover, it inspires reluctant learners to participate in the discussion ( Keogh & Naylor, 1999). Concept animation proposes the enhancements to the concept cartoon or graphic medicine’s educational approach and fosters a shift in student’s knowledge from cognitive to metacognitive level.

## Concept Cartoon and Concept Animation

Concept animation is aligned with the ideology and approach of concept cartoon in fostering students understanding of basic and advanced level concepts of health sciences. The interaction and dynamics of the comic characters supplemented by speech balloon may raise students’ curiosity of a concept and advance him further for a problem-solving approach. Likewise, it may also help to clarify the misconception that might have arisen from their lack of understanding of the textual information. It has a contextual relevance in Middle Eastern countries where national curriculum treats the English language more of a subject than a medium of wider communication for local and global uses. This situation made the teacher to employ concept animation as a substitution of communicating ideas which were not easy to learn by textual information only. Furthermore, it may facilitate students’ interest and their level of motivation in a similar way as concept cartoon (Naylor & Keogh, 2013).

## Theoretical framework

**Figure 1.**
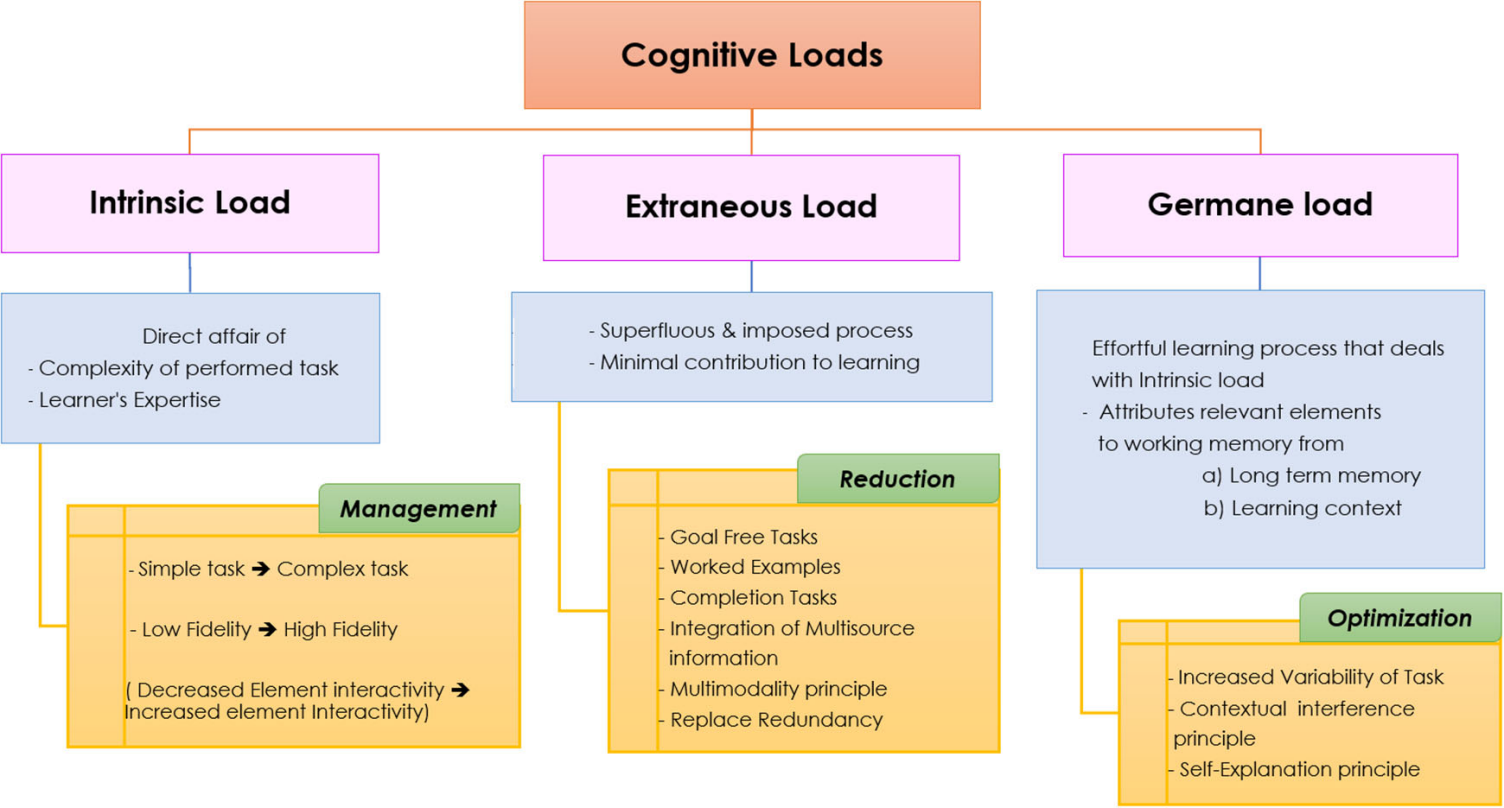
Schematic representation of different cognitive loads, brief description and principles recommended by cognitive load theory

**Figure 2.**
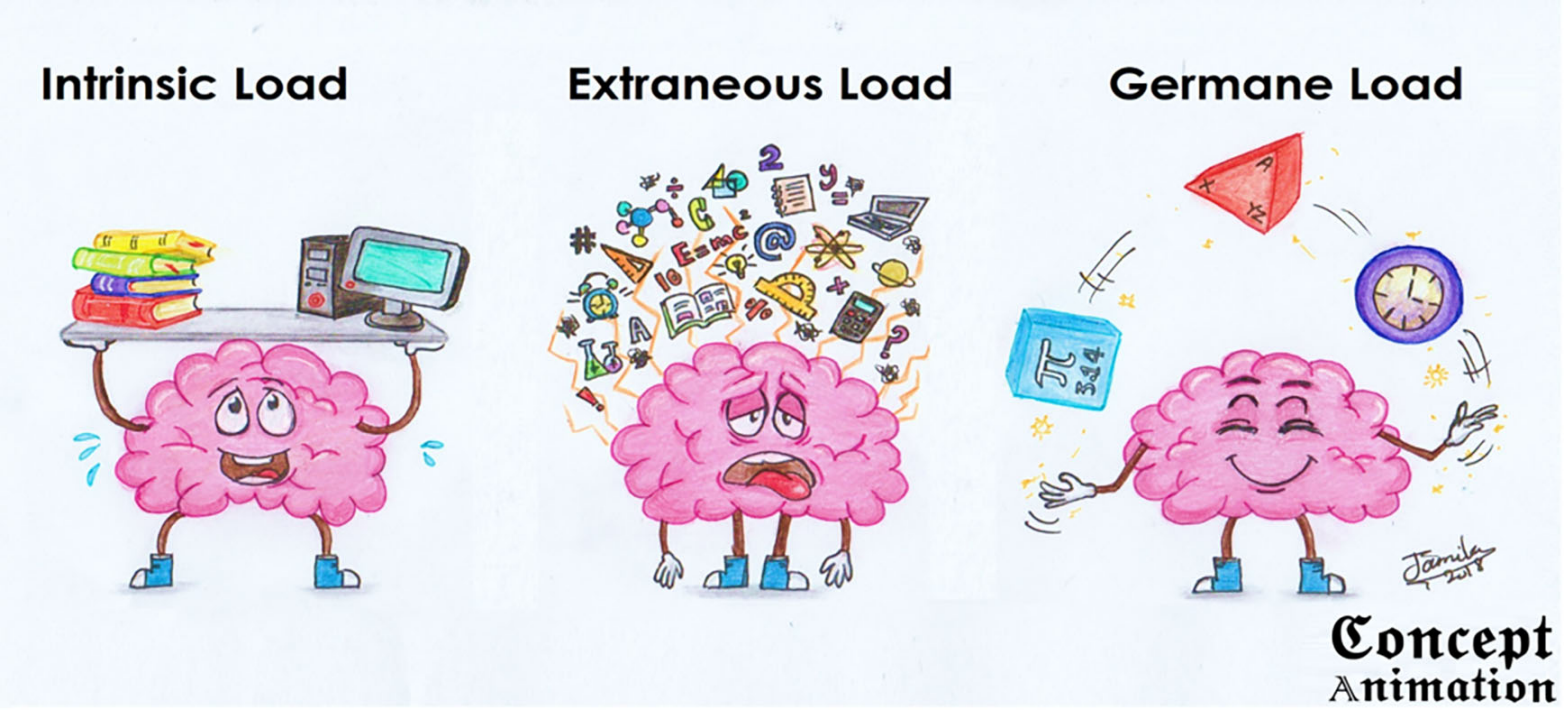
Comic illustration of different cognitive loads

a. **Instructional scaffolding and constructivism**
  Concept animation is an extravaganza of unifying images in sequence as visual clues complemented by texts as supportive information unveiling story/stories. It can serve as an instructional scaffolding described by Vygotsky- the teacher guided, series of small steps to extend an existing schema of prior knowledge into the new cognitive domain. It can operate as a tutor guided problem-solving strategy to improve the level of potential development among student ( Vygotsky, 1978).
  As described by Vygotsky the ‘zone of proximal development’ lies between actual and potential developmental levels, where actual signifies independent problem-solving aptitude and potential represents the development of problem-solving skills under tutor guidance or with more competent peer collaboration. It involves the development of a mutual understanding of teacher and student ideas once the extension is constructed. Later the tutor can withdraw his guidance, providing the student with the opportunity to independently use the newly acquired problem-solving skills (Cakir, 2008).
  The concept animation strategy proposed to teach core and advanced level concepts can facilitate students understanding by visually reconnecting them with their prior knowledge. Furthermore, the animation may offer them ‘visual clues’ to prompt their independent problem-solving skills in the zone of potential development. Thus, concept animation can function as a scaffold supporting the student during their learning of concepts and acquisition of independent problem-solving skills through the facilitation of a competent teacher or knowledgeable peer.
b. **The cognitive theory of multimedia**
  According to the cognitive theory of multimedia learning, the learner needs support and guidance for cognitive processing of information. The learning should focus on essential and generative processing; essential cognitive processing requires to mentally display vital information from a lesson stored in the working memory. However, generative processing fosters integration and organization of presented material; in our case ‘comics ‘and ‘animations’ which is driven by learner’s motivation to understand concepts ( Mayer, 2010).
  Concept animation can complementarily endorse an effective instructional design to encourage essential and generative processing among students in achieving meaningful learning outcomes. The cognitive theory of multimedia learning emphasizes that for meaningful learning to occur, learners should be engaged in relevant cognitive processing such as concentrating on relevant material, mentally establishing the information into a coherent cognitive display and integrating it with prior knowledge already stored as long-term memory ( Mayer, 2010).
  Concept animation makes effective use of dynamics shown among static images of comic and cartoon characters to stimulate and reinforce students essential and generative processing of information respectively for a meaningful learning experience. Student’s interest in concept amination can serve as driving force for their motivation that can eventually transform learning into a meaningful worthwhile experience. Comics as graphic medicine tool has been acknowledged as an effective medium for students studying anatomy; it fosters student interest and motivation in the subject ( Kim, Chung, Jang & Chung, 2016). Conversely, graphic medicine and concept animation has not been hugely adopted as a multimedia strategy to transform teaching/learning behaviour of students.
c. **Cognitive load theory**
  Cognitive load theory aspires the guidelines for the development of instructional frameworks based on a model of human cognitive architecture. According to it the working memory of the human cognitive system is extremely limited; the limitations are exclusively applicable to novel information acquired through sensory memory. Albeit, working memory faces no known limitation handling information retrieved from long-term memory. Long-standing memory endures information as cognitive schema; this offers learners the ability to classify multiple elements of information as single element improving the capacity of working memory for learning. Expertise evolves as learners assimilate simple schema to more complex ones. The learning is facilitated by the construction and automation of such cognitive schemas; the automation helps to liberate working memory capacity for other activities. A well designed instructional layout should not only endorse schema construction but also schema automation for tasks of consistent nature ( Van Merrienboer & Sweller, 2010). Figure 1 details the schematic representation of different cognitive loads with a brief overview. Figure 2 shows the comic illustrations of the cognitive loads.

## Concept Animation in undergraduate teaching

Technical and allied health sciences students consider basic sciences courses as a ‘hurdle to jump over’ to get into the real, skill-oriented clinical content. There is an imperative need to integrate basic sciences courses using conceptual streams for advanced training of allied health sciences students. These streams ensure a smooth and continuous flow of concept development along the entire duration of the study leading to a progressive increase in complexity, analysis and expectations ( Taggart & Sanchez, 2016). Keeping this idea in mind, concept animation was employed to communicate the advanced level concepts of a biosciences related course of pharmacology to undergraduate optometry student.

The fundamental concepts of drug pharmacokinetics, pharmacodynamics, side effects and bacterial resistance against antibiotics were transformed into comics. The apparent and interactive activities of the comic characters as series of diagrams tend to facilitate students understanding of the core concept of the subject. The basic concepts were represented by diagrams and accompanied by text in speech balloons. These speech balloons enclose essential but obscure information as clues. These self-explanatory clues may direct student understanding of concept development by prompting their generative processing. The functional model of generative learning contributes to effective instructional methodologies that often yield substantial gain in comprehension and understanding ( Wittrock, 1992).

**Figure 3.**
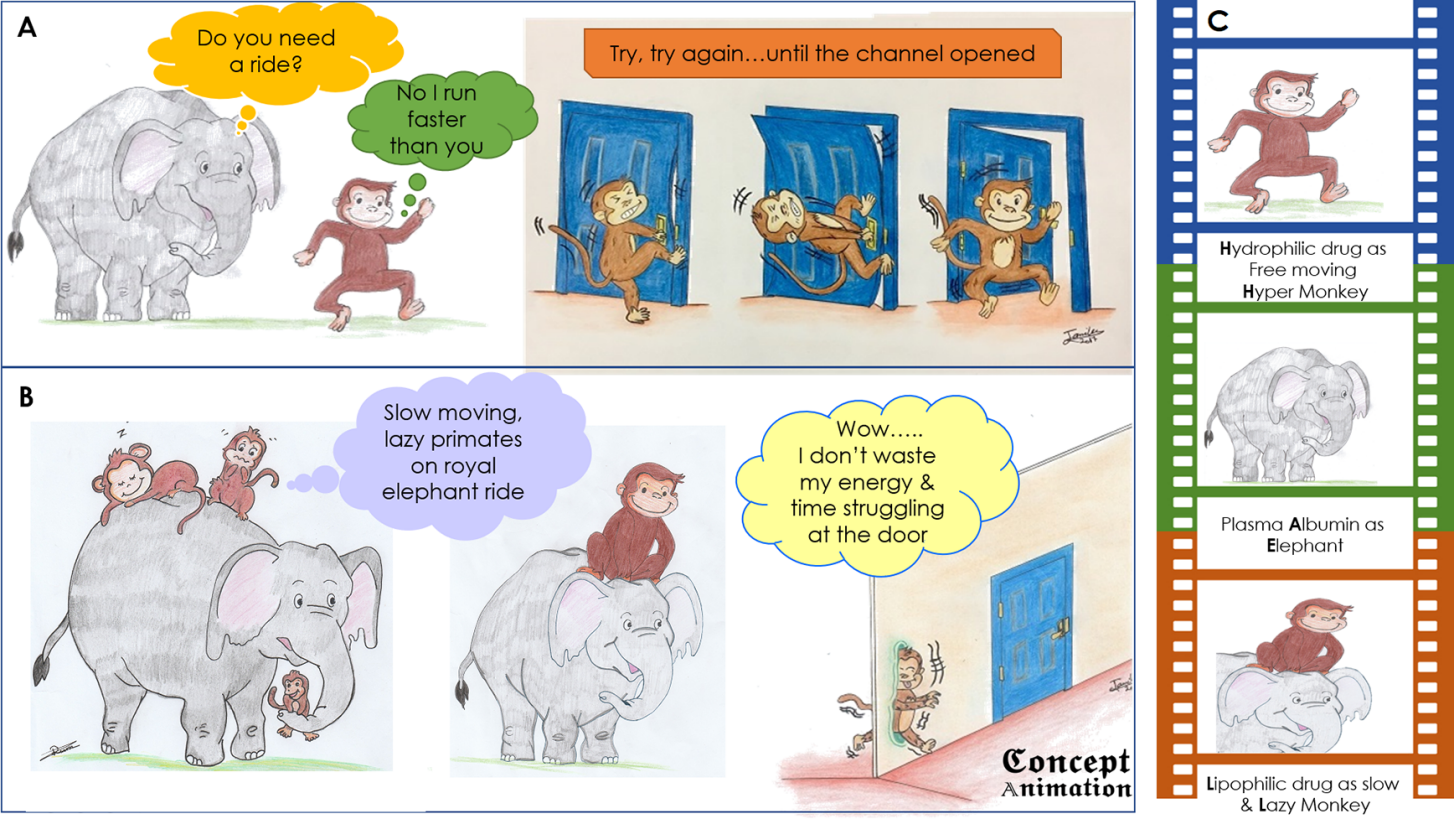
Pharmacokinetic comic of hydrophilic (A) and lipophilic drugs (B); a brief portfolio of the characters is given as a photographic film (C) on the right side

The pharmacokinetic comics of hydrophilic and lipophilic drugs shown in Figure 3 has two important characters, elephant and monkey; where elephant being a large mammal is compared with plasma protein Albumin while an analogy is made between monkey and drugs (hydrophilic and lipophilic drug). For instance, in the upper segment of the comic (A), the monkey does not take a ride on the elephant, as it is active and free moving like a hydrophilic drug in plasma. However, after reaching the target site (cell), it struggles a lot to enter and finally, a door (channel protein) opens allowing the monkey to go inside as shown in the succeeding part of the comic.

The lower segment (B) shows slow, lazy monkeys sitting on the elephant as indolent species; after reaching the destination, the lazy monkey does not bother to open the door, it crosses the wall through and through without any struggle; depicts the pharmacokinetic behaviour of many of the lipophilic drugs. Furthermore, this comic made the student brainstormed many aspects of drug interaction and dynamics, ultimately guiding the cognition to the zone of proximal development. It made the students inquire about the interaction and bonding between lipophilic drugs and plasma protein albumin, thus encouraging them to engage in the generative processing of the information provided as visual clues. It reinforced their understanding of the physicochemical properties of the cell membrane that made a lipophilic drug to easily cross and hydrophilic drug to struggle a lot. Interestingly, these comics made them inquire and learn concepts which were not entirely obvious by the diagrams such as volume of drug distribution (V _D_ ) and drug depot effect etc using the self-explanation principle of optimizing germane load.

**Figure 4.**
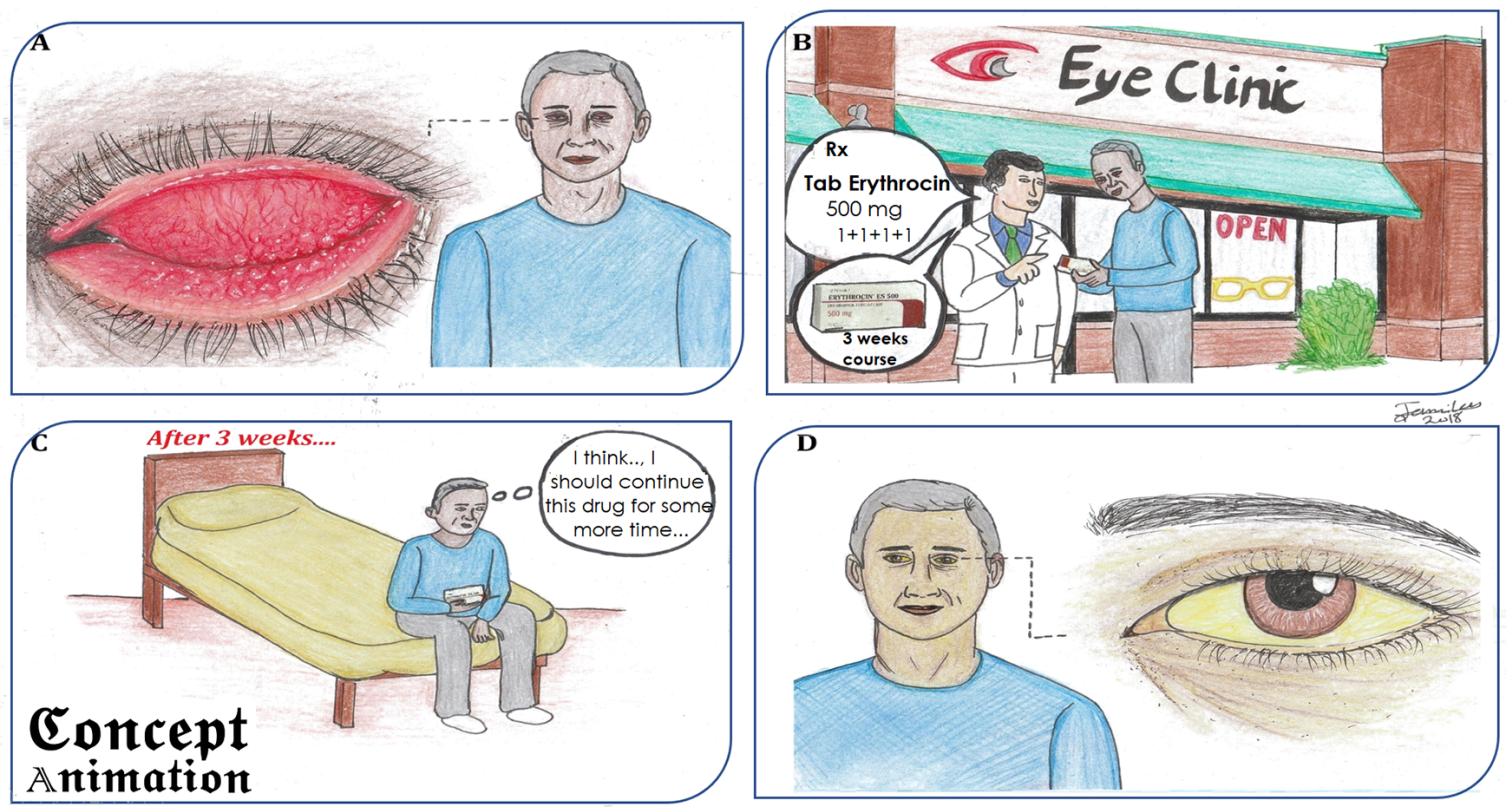
Concept animation showing dose-related toxicity of antibiotic ‘Erythromycin’

Likewise, the advanced level concept of drug dose-related toxicity of antibiotic such as ‘Erythromycin’ is visually supported by the comic shown in Figure 3. The patient with a severe form of conjunctivitis ‘inclusion conjunctivitis’ visited an eye clinic ( Figure 4A). The doctor advised him to use tablet Erythromycin 500 mg, four times a day (QID) for 3 weeks ( Figure 4B). After 3 weeks of treatment, the patient decided to continue the drug for some more time that could result in Erythromycin ‘dose-related toxicity ( Figure 4C) This toxicity is the outcome of cholestasis precipitated by increased drug concentration or decreased hepatic level of anti-oxidants such as glutathione. It may present with or without jaundice and other non-specific symptoms weeks or months after the treatment ( Padda, Sanchez, Akhtar & Boyer, 2011). In the last segment ( Figure 4D) of the comic, the same patient is shown with yellow discolouration of the eye (sclera) that is the manifestation of the adverse dose-related effect of the drug.

## Facilitative Effects of Concept Animation

Concept animation helped students to develop consistency, uniformity and homogeneity of ideas towards the understanding of the integral concepts of the subject. It offered them a strategy to construct their knowledge and reinforce their understanding using visual aids such as comics, stories and animation. This approach can evolve students’ interest and inclination in the discipline. It encompasses the cognitive range from the lower order to higher order thinking skills. It can function as an effective substitution of information redundancy and overload. Concept animation can foster student metacognitive abilities and imagination.

Concept animation functions as the visual metaphor as it utilizes shape and components of familiar natural or manmade objects or of a simply recognizable story. The meaningful organization and association of content within the metaphor convey additional information about the content. The level of their difficulty is low to medium, with high understandability ( Eppler, 2006). Concept animation facilitates the process of knowledge transfer and learning as it supports students as a ‘potential instructional scaffolding’ to connect what they already know with new information.

Furthermore, Concept animation can help in reducing extraneous load, managing the intrinsic load and optimizing generative load as recommended by the cognitive load theory shown in Figure 1. For instance, the redundancy principle recommends the replacement of multiple, redundant source of information with the only single source of information ( Van Merrienboer & Sweller, 2010). Concept animation can work on these principles in reinforcing student’s motivation towards generative processing of the information.

Presenting solution with a diagram can facilitate student learning and minimize the split attention as the concept is presented with the single source of information (Agostinho, Tindall-Ford & Bokosmaty, 2014). Concept animation can reduce the split attention of the learners as illustration and discussion are integrated into a single entity.

The questionnaire survey performed in an earlier study showed an acknowledgement of 85% of students towards ‘concept animation’; they found it as an effective strategy that helped them understand the difficult concepts in an easy way ( Khurshid & Noushad, 2017).

## Limitations

Concept animation has a selective approach, as not all concepts can be flexibly communicated by animations; sometimes it can divert or distract students from the actual content if not carefully and precisely designed. It can only be utilized for the communication of basic or advanced level concepts depending on the learning aptitude and the intelligence level of the students. The comics drawing is a time-consuming process, as it needs a substantial amount of work, skill and energy.

## Take Home Messages

Concept animation is as a multimedia approach to learning that can direct the student to process the knowledge utilizing their imagination and generative processing in the constructive understanding of integral concepts ranging from fundamental to advanced levels.

## Notes On Contributors

FARAZ KHURSHID, MBBS, MS Molecular Biology, MHPE, is a Lecturer at College of Health Sciences, University of Buraimi, Oman. His enduring relationship with academics and relevant research skills encompassing from basic sciences discipline, molecular biology, to health professions education. His research interests include metacognition, threshold concepts, multimedia learning and learning environment.

BABU NOUSHAD is a Lecturer of Optometry at the College of Health Sciences, University of Buraimi, Sultanate of Oman. He has master’s degrees in optometry and Health Professions Education. His research interests include learning environment and instructional designs.

INGRID A. E. SPANJERS, PhD, is a teacher at the Graduate School of Health Professions Education (SHE), Maastricht University, Maastricht, the Netherlands. She has a background in general and special education. Her research interests are multimedia learning, animations and cognitive load theory.

JAMILA AL-DARWASHIis an undergraduate final year student of Optometry at College of Health Sciences, University of Buraimi, Oman. She is one of the brightest students with a good academic standing. She is innovative and parallelly uses her talent in drawings with her own set of innovative ideas.
