# Supplementary material for: Concept Animation - a potential instructional scaffolding
Source: MedEdPublish (2016). 2018 Jul 24;7:153. [Version 1] doi: 10.15694/mep.2018.0000153.1 (PMC10701852; doi:10.15694/mep.2018.0000153.1)
Supplement: Peer review history

# Reviewer report: Reviewer response for version 1

**DOI:** 10.21956/mep.19609.r29142

**Published:** 2019-02-25

**Authors:** Trevor Gibbs

**Affiliations:** 1 AMEE

This review has been migrated. The reviewer awarded 3 stars out of 5 An interesting paper about an interesting active learning idea. I am afraid that after reading this paper however and looking at the animation examples used, I am still left rather sceptical as to whether the value of this form of learning exceeds the novelty shown. I suppose we all do this to some extent in our PowerPoint or other types of presentation- some audiences enjoy it whilst others are still left confused. I hope the authors can follow this paper up with a more long term evaluation that perhaps reverses my opinion.

Reviewer Expertise:

NA

No decision status is available

# Reviewer report: Reviewer response for version 1

**DOI:** 10.21956/mep.19609.r29141

**Published:** 2018-11-02

**Authors:** Rhoda Muliira

**Affiliations:** 1 University of Buraimi

This review has been migrated. The reviewer awarded 5 stars out of 5 In higher education teachers prefer using visual aid such as diagrams, posters and lots of YouTube videos these days to help students retain information. I want to commend the authors for initiating an effective teaching strategy for adult learners. Using animations as visual aid for advanced learners is an innovative strategy to help them retain information especially for a subject like pharmacology which is sometimes challenging to master. I think this strategy may even increase the "20% of what we see we remember" to a higher value because of the interesting way that concepts are presented. I highly recommend other educators to try and use this innovative strategy of visual learning in their teaching.

Reviewer Expertise:

NA

No decision status is available

# Reviewer report: Reviewer response for version 1

**DOI:** 10.21956/mep.19609.r29138

**Published:** 2018-08-12

**Authors:** sadaf asif

**Affiliations:** 1 not applicable

This review has been migrated. The reviewer awarded 5 stars out of 5 This article covers aspects on which there is not a big body of research. Some research was done in the 80s which showed hat it was beneficial for some areas for some of the time.This is a relatively clandestine area of research. Although animation has been viewed as a legitimate form of literature owing to their powerful visual messages that convey immediate visceral understanding in ways that conventional texts cannot; However, due to preconceived bias about its application in learners creates barrier in learning.In keeping with research in medical education which shows that visual art improves students’ diagnostic skills and may enhance students’ observational and interpretive abilities; it could cultivate awareness of the broader social and political issues within Medicine. Research can help to dispel doctors’ scepticism about the use of graphic stories as few empirical studies exist on their effect in medicine. Future research from a wide range of disciplines might focus on ways to show the effectiveness of this tool in medicine. Lastly, this article will help in developing a full fledged research on this type of learning as before, traditional ways of learning has created barriers for visual learners who would have been gifted in way that don't fit the traditional model of learning. We need more research as to the reasons outlined above.

Reviewer Expertise:

NA

No decision status is available

# Reviewer report: Reviewer response for version 1

**DOI:** 10.21956/mep.19609.r29139

**Published:** 2018-07-29

**Authors:** Mazoun Musallam

**Affiliations:** 1 University of Buraimi,HOS,optometry

This review has been migrated. The reviewer awarded 5 stars out of 5 It’s a great idea . We used to see illustration as “cartoon”for school student. l still remember majority of the animation illustrations in the active school class . This type of teaching is more effective and efficient technique To save the information in long memory ,also it breaks the routine of teaching. In addition to that it give the lecturer more space in teaching and add same fun to the medical lecture .��I like it , and l wish all the best for ur team and this active idea .

Reviewer Expertise:

NA

No decision status is available

# Reviewer report: Reviewer response for version 1

**DOI:** 10.21956/mep.19609.r29140

**Published:** 2018-07-29

**Authors:** Nasser Mohammed

**Affiliations:** 1 University of Buraimi

This review has been migrated. The reviewer awarded 5 stars out of 5 It's great idea .lt will be helpful for students.This idea proves the information and makes the learner connect all ideas they have. I also believe that this idea goes hand in hand with the age of technology we live in.

Reviewer Expertise:

NA

No decision status is available

# Reviewer report: Reviewer response for version 1

**DOI:** 10.21956/mep.19609.r29143

**Published:** 2018-07-24

**Authors:** P Ravi Shankar

**Affiliations:** 1 American International Medical University

This review has been migrated. The reviewer awarded 4 stars out of 5 This is an interesting article about concept animation and scaffolding. The authors describe the use of concept animation in pharmacology learning. The examples they have provided illustrate the concept well. Creating comics will require good drawing and creative skills as mentioned by the authors. The visual nature and identification with the characters can lead to improved understanding and retention. ‘Comic books’ could be created and made commercially available to help faculty and students with different topics. I enjoyed reading this article which will be of interest to a wide group of medical educators.

Reviewer Expertise:

NA

No decision status is available
